# Dawn to Dusk: Diurnal Rhythm of the Immune Response in Rainbow Trout (*Oncorhynchus Mykiss*)

**DOI:** 10.3390/biology9010008

**Published:** 2019-12-30

**Authors:** Ruth Montero, Joanna Ewa Strzelczyk, Justin Tze Ho Chan, Marieke Verleih, Alexander Rebl, Tom Goldammer, Bernd Köllner, Tomáš Korytář

**Affiliations:** 1 Friedrich-Loeffler-Institute, Federal Research Institute for Animal Health, Institute of Immunology, 17493 Greifswald-Insel Riems, Germany; ruth.montero.m@gmail.com (R.M.); jnnjaros@gmail.com (J.E.S.); bernd.koellner@fli.de (B.K.); 2 Institute of Parasitology, Biology Centre of the Czech Academy of Sciences, 37001 České Budějovice, Czech Republic; justin.chan@paru.cas.cz; 3 Leibniz Institute for Farm Animal Biology (FBN), Institute of Genome Biology, 18196 Dummerstorf, Germany; verleih@fbn-dummerstorf.de (M.V.); rebl@fbn-dummerstorf.de (A.R.); tom.goldammer@uni-rostock.de (T.G.); 4 Faculty of Fisheries and Protection of Waters, University of South Bohemia, 37001 České Budějovice, Czech Republic

**Keywords:** diurnal rhythm, blood composition, myeloid cells, phagocytic cells, chronoimmunology

## Abstract

The daily change of light and dark periods influences different physiological processes including feeding, resting and locomotor activity. Previously, several studies on mammalian models revealed a strong link between day-night rhythms and key immunological parameters. Since teleost fishes possess innate and adaptive immune responses like those observed in higher vertebrates, we aimed to elucidate how changes in light-dark cycles shape the immune system of fish. Using the rainbow trout laboratory model, we investigated the link between diurnal rhythms and immune competence of fish. Initially, the cell composition and phagocytic activity of leukocytes was analyzed in the circulation as well as in the head kidney, the functional ortholog of mammalian bone marrow. Once the baseline was established, we evaluated the ability of fish to respond to a bacterial stimulus, as well as the changes in antimicrobial activity of the serum. Our results suggest increased immune competence during the day, manifested by the higher presence of myeloid cells in the circulation; increased overall phagocytic activity; and higher capacity of the sera to inhibit the growth of *Aeromonas salmonicida*. Notably, our flow cytometric analysis identified the myeloid cells as the major population influenced by the time of day, whereas IgM^+^ B cells and thrombocytes did not vary in a significant manner. Interestingly, the presence of myeloid cells in blood and head kidney followed complementary trends. Thus, while we observed the highest number of myeloid cells in the blood during early morning, we witnessed a reverse trend in the head kidney, suggesting a homing of myeloid cells to reservoir niches with the onset of the dark phase. Further, the presence of myeloid cells was mirrored in the expression of the proinflammatory marker *tnfa* as well as in the number of leukocytes recruited to the peritoneal cavity in the peritonitis model of inflammation. Overall, the data suggest a connection between diurnal rhythms and the immune response of rainbow trout and highlight the relevance of rhythmicity and its influence on experimental work in the field of fish chronoimmunology.

## 1. Introduction

Daily rotation of the planet has been the major driving force of the evolution of circadian rhythms [1]. Initially evolving to protect anaerobic bacteria from the toxic effect of oxygen, this mechanism was adopted by living creatures and influences a plethora of physiological processes. In vertebrates, 24-h rhythms are controlled by the suprachiasmatic nucleus (SCN) in the hypothalamus which coordinates the activities of various peripheral oscillators and regulates rhythmic physiological processes such as locomotion or hormonal secretion [2]. At molecular level, the inner clock consists of a well-defined set of transcription factors intertwined in the auto-regulatory feedback loops [3]. These rhythms are entrained to environmental cues including external light, which for example influences the secretion of melatonin by the pineal gland [4]. Unlike mammals, which exhibit very stable and constant rhythms, the circadian system of fishes can be diverse and easily influenced by the environmental conditions, feeding habits and danger of predation. [5]. Thus, while some fishes such as zebrafish (*Danio rerio*) [6] or tropical Yellow Wrasse (*Halichoeres chrysus*) [7] exhibit clear daily rhythms of activity, some species show great inter-individual variability [8]. Salmonids can be active at day and night, demonstrating more flexibility in the circadian rhythms compared to other fish families [5].

Numerous studies have provided evidence of a strong connection between circadian rhythms and immune system, since several key parameters have been described to be dependent on the time of day. This includes the number of haematopoietic cells and circulating leukocytes; the level of hormones and cytokines [9,10,11]; phagocytic activity [12]; cytotoxic activity of natural killer cells [13]; antigen presentation, proliferation [14,15,16] and humoral immune responses in lymphocytes [17]. Furthermore, circadian rhythms were shown to influence the susceptibility to infections and the course of diseases [18,19]. Although the majority of these findings were described for mammalian species, we expect that circadian regulation of the immune response is deeply embedded in evolution and is a determinant of the immune responses of early vertebrates as well. The rainbow trout (*Oncorhynchus mykiss*) is a useful model for understanding basal vertebrate immune systems. As in higher vertebrates, they mount innate and adaptive responses executed by lymphoid and myeloid cells, and are orchestrated by cytokines and chemokines [20]. At the same time, fish lymphocytes often show innate features, which are largely exclusive to populations of innate-lymphocytes, B1 B cells, or γδ T cells in higher vertebrates [21,22,23]. In the present study, we use this animal model to elucidate how the immune competence of fish mirrors changes in light/dark periods of the day.

## 2. Materials and Methods

### 2.1. Fish

Rainbow trout (*Oncorhynchus mykiss*) of the commercially available strain TCO Steelhead II (Tacoma, USA) was obtained from the *State Research Centre for Agriculture and Fishery (LFA-MV), Institute of Fishery, Born, Germany*. The juveniles were transported to the facility in Czech Republic, were they were kept for over 6 months prior to the experiment. No diseases or mortalities were recorded before and during the experiment. Animals were kept in 1000 L tanks at 15 °C ± 1 °C in partially recirculating water systems and were fed twice a day with commercial dry pellets. The light period was fixed to 12L:12D, starting at 6:00 and ending at 18:00. Animal procedures were performed in accordance with Czech legislation (section 29 of Act No.246/1992 Coll., on Protection of animals against cruelty, as amended by Act No. 77/2004 Coll.) and animal handling complied with the relevant European guidelines on animal welfare (Directive 2010/63/EU on the protection of animals used for scientific purposes) and the recommendations of the Federation of Laboratory Animal Science Associations. The animal experiments have been approved by the Ministry of Education, approval ID: MSMT-18301/2018-2. For the experiment, a total of 20 fish were used for the analysis of cell composition, the phagocytic assay and the gene expression analysis. Additional 20 fish were used for the in vivo stimulation experiment with the inactivated *A. salmonicida*.

### 2.2. Sampling and Leukocyte Preparation

Blood was collected from the caudal vein using heparinized syringes and immediately diluted in mixed cold medium (Iscove’s Dulbecco’s modified Eagle’s medium (DMEM) and Ham’s F12 (Gibco, Germany) at a ratio of 1:1). Head kidneys were homogenized to prepare single cell suspensions. The peritoneal leukocytes (PEL) were obtained via lavage with 5 mL ice-cold 1x PBS containing 5 mM EDTA (Thermo Fisher Scientific, Pardubice, Czech Republic).

### 2.3. Flow Cytometry

For the analysis of cell numbers and distribution of lymphoid and myeloid populations, 5 fish per time point were euthanized. Isolated peritoneal leukocytes were resuspended in 1 mL total of 1x phosphate-buffered saline (PBS)-1mM EDTA. 100 µL of cell suspension was diluted in 300 µL of 1x PBS-1mM EDTA. Cells were acquired on a FACS Canto II (BD Biosciences, Prague, Czech Republic) on high throughput for 20 s. The number of cells was counted with an equation based on measurement of a dilution row of head kidney leukocytes.

The analysis of cell composition was performed using multicolour flow cytometry with a set of rainbow trout-specific monoclonal antibodies: anti-thrombocytes (MAb42-1 [24]), anti-IgM^+^ B cells (MAb 1,14 [25]); anti-myeloid cells (MAb21). The sample preparation was performed as follows: 10 µL of full blood or head kidney cell suspensions were incubated for 30 min at 4 °C with directly labelled antibodies as described previously [26].

### 2.4. Phagocytosis of Latex Beads

To evaluate the phagocytotic potential of the blood leukocytes, 100 µL of blood was mixed with 5 µL of fluorescent latex beads (1 µm) (Sigma). Cells were incubated for 2 h at 15 °C in 2.5% CO_2_. Following incubation, cells were washed twice with 1x PBS-1mM EDTA and resuspended in 150 µL. In the final step, cells were stained for 20 min with the mixture of directly labelled antibodies recognizing IgM^+^ B cells, myeloid cells and thrombocytes. After a final wash, cells were measured on FACS Canto II (BD Biosciences, Prague, Czech Republic).

### 2.5. Aeromonas salmonicida for Stimulation Experiments

The *Aeromonas. salmonicida* ssp. *salmonicida* wild-type strain JF 2267 used for all stimulation trials was kindly provided by J. Frey, University of Bern, Switzerland. The bacteria were cultivated from cryoconserved batches (MicrobankTM, PRO-LAB Diagnostics) on casein-peptone soymeal-peptone (CASO) slant agar (lysogeny broth (LB); SIFIN) at 15 °C for 98 h and subsequent mass culture replant in LB broth (SIFIN) for another 48 h. The initial cultures were checked for purity by Gram staining, cell morphology and motility. The bacterial suspension was concentrated by a 10-min centrifugation step at 4000 g at 4 °C. The supernatant was discarded and the bacterial pellet was washed once in sterile 0.9% sodium chloride solution and diluted to 1 x 10^8^ bacteria/mL. Each dose was controlled afterwards by counting the colony-forming units (CFU) after culturing the bacteria on CASO agar plates at 15 °C.

For stimulation experiments, bacteria were inactivated in 1.5% paraformaldehyde for 1 h. The suspension of inactivated *A. salmonicida* (i*A.s.*) was adjusted to a concentration of 1.6 × 10^9^ cells/mL, frozen in 1 mL aliquots and kept at −20 °C. Prior to use, the bacteria were thawed and diluted to the concentration of 5 × 10^7^ cells/mL in sterile 1x PBS.

### 2.6. In Vivo Stimulation Experiment

Rainbow trout (*n* = 5 per group) received a single intraperitoneal (i.p.) injection of 200 µL containing 1 × 10^7^ i*A.s*. Fish were sampled 6 h post-injection (hpi) and peritoneal leukocytes were isolated as described above.

### 2.7. In Vitro Stimulation Experiment

To evaluate the ability of head kidney leukocytes to mount a proinflammatory response, 2 × 10^6^ cells were stimulated with 2 × 10^6^ units of i*A.s*. Six hours post-stimulation, cells were collected and RNA was prepared using the RNeasy kit (Qiagen, Bustehrad, Czech Republic) according to the manufacturer’s instructions. The expression of the proinflammatory cytokines IL1β and TNFα, and the chemokine *cxcl12* and its receptor *cxcr4*, were analyzed by quantitative real-time polymerase chain reaction (qRT-PCR) using primer pairs for the target genes described previously [27]. The qRT-PCR was performed on the CFX96 Touch™ Detection System (Bio-Rad, Feldkirchen, Germany) using SensiFAST^TM^ SYBR one-step kit (Bioline, Heidelberg, Germany) according to the manufacturer’s protocol. To assess the specificity of the PCR amplification, a melting curve analysis was performed at the end of each run.

### 2.8. Bacterial Growth in Serum

Blood for serum preparation was collected at 6:00, 12:00, 18:00, and at 24:00 (24-h system). Once clotted, serum was obtained by centrifugation at 3000 g for 10 min at 4 °C. The supernatant was collected and stored at −20 °C.

For the bacterial growth assay, 100 µL of serum (two technical replicates) from each fish was inoculated with 2 × 10^5^ bacteria *A. salmonicida* ssp. *salmonicida* strain JF2267. Sera with bacteria was incubated for 24 h at 18 °C. After incubation, the bacteria culture from each well was centrifuged at 4000g for 10 min at 4 °C. Supernatant was discarded and total DNA was isolated from each pellet using QIAamp DNA Blood Mini Kit (Qiagen, Hilden, Germany) according to the manufacturer’s protocol.

The number of bacterial cells that grew over 24 h was quantified on the CFX96 Touch™Detection System (Bio-Rad, Feldkirchen, Germany) using SensiFAST^TM^ SYBR one-step kit (Bioline, Heidelberg, Germany) according to the manufacturer’s protocol. To verify the quality of the PCR product, melting curve analysis were performed at the end of the run. The number of cells was calculated on the basis of a standard curve prepared by using DNA from serial 10-fold dilutions of bacteria, ranging from 1 × 10^2^ -1 × 10^8^ cells.

### 2.9. Statistical Analysis

The data was analyzed using the software GraphPad Prism 8 for Mac (San Diego, CA, USA). In the graphs, the data is presented as mean values ± standard deviation (SD). The statistical significance was assessed by one-way analysis of variance (ANOVA) and the Bonferroni *post hoc* test.

## 3. Results

### 3.1. Cell Composition of Blood and Head Kidney Leukocytes

**Figure 1:**
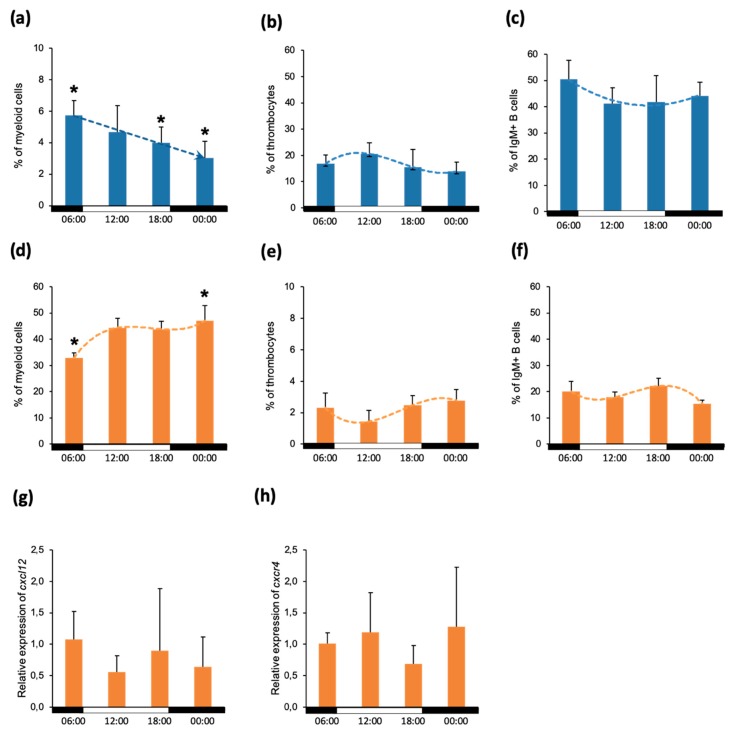
Composition of peripheral blood and head kidney leukocytes. Proportion of myeloid cells (**a**), thrombocytes (**b**) and IgM+ B lymphocytes (**c**) in peripheral blood. The composition of head kidney is shown in the second row of the graphs: myeloid cells (**d**), thrombocytes (**e**) and IgM+ B cells (**f**). Graphs (**g**) and (**h**) represent the relative expression of the genes *cxcl12* and *cxcr4*, respectively. The data is presented as mean values with standard deviation (SD) error bars. The statistical analysis used was one-way analysis of variance (ANOVA) and the Bonferroni *post hoc* test with *p* ≤ 0.05. Statistical differences are represented by asterisks (*). The white and black bars on the x-axis demonstrate the dark/light period.

In mammalian models, it has been demonstrated that the time of day strongly influences the composition of the major leukocyte subsets in circulation and in primary lymphoid organs. To elucidate this phenomenon in fish, we analyzed the cell composition of the peripheral blood and head kidney, a fish ortholog of the bone marrow. For this, five fish were sampled every 6 h (6:00, 12:00, 18:00, and 24:00) and the cell composition was analyzed using multicolour flow cytometry. Results from peripheral blood suggested only marginal differences in the ratio of erythrocytes and leukocytes ranging from around 330,000 to 420,000 erythrocytes: 10,000 lymphocytes at all measured time points (data not shown). As for the composition of leukocytes, the most prominent differences were observed in the population of myeloid cells, which was highest at 6:00 (6.8%) and declined significantly through the day down to 3% at midnight (Figure 1a). The percentage of thrombocytes ranged from 16%–20%, reaching the highest value at 12:00 (Figure 1b) and the proportion of IgM^+^ B cells ranged between 41% and 51% with a mild decline throughout the day (Figure 1c).

In the head kidney, cells of myeloid origin represented the major population, ranging between 35% and 50%. Notably, the lowest percentage of myeloid cells was observed in the early morning and their number increased significantly in the later hours of the day (Figure 1d). The number of the thrombocytes was low and ranged from 1.5%–3% without significant differences between the time points (Figure 1e). Similarly, the percentage of IgM^+^ B cells present in the head kidney varied between 15% and 22% with no clear trend throughout the day (Figure 1f).

In mammals, the recruitment of the leukocytes into the blood was demonstrated to be dependent on the expression of the homing chemokine CXCL12 by the stromal cells and the presence of its receptor CXCR4 on the surface of leukocytes. To this end, we aimed to analyze the expression of these genes in the cells isolated from the head kidney. Despite the mild fluctuations in the expression of both genes observed throughout the day, no significant difference was observed (Figure 1g,h).

### 3.2. Phagocytic Activity of Blood Leukocytes

**Figure 2:**
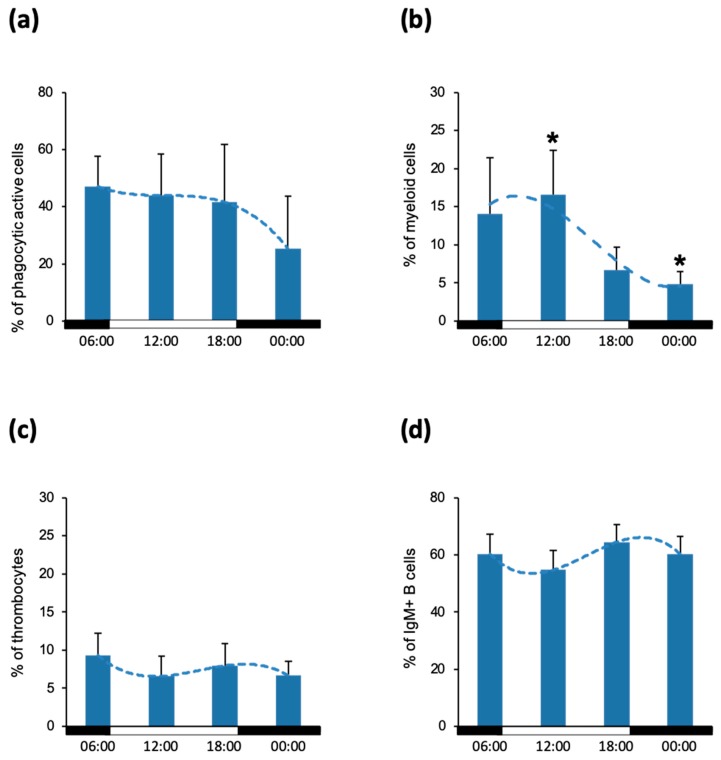
Phagocytic activity of peripheral blood leukocytes. Proportion of cells taking up fluorescent latex beads in the population of whole blood (**a**), myeloid cells (**b**), thrombocytes (**c**) and IgM^+^ B cells (**d**). The data is presented as mean values with SD error bars. The statistical test used was one-way ANOVA with the Bonferroni *post-hoc* test. *p* ≤ 0.05 was considered statistically significant and indicated by asterisks (*). The white and black bars on the x-axis represent the dark/light period.

Next, we investigated whether the time of day is a determinant in the capacity of leukocytes to phagocytose particles. Here, peripheral blood leukocytes were tested for uptake of fluorescent latex beads. The obtained results suggest that the percentage of phagocytic active cells during the day ranged between 25% and 57%. Notably, the proportion was highest in the morning (57%) and decreased towards the evening and night, when it eventually dropped down to 25% (Figure 2a). Interestingly, myeloid cells represented only 5%–17% of total phagocytic active cells (Figure 2b), having a peak at midday and decreasing afterwards, while the IgM^+^ B cells were the major population of phagocytes present in the blood (Figure 2d) in a constant proportion through the day. Lastly, the thrombocytes accounted for 6%–10% of phagocytic cells (Figure 2c).

### 3.3. In Vitro Stimulation of Head Kidney Leukocytes

**Figure 3:**
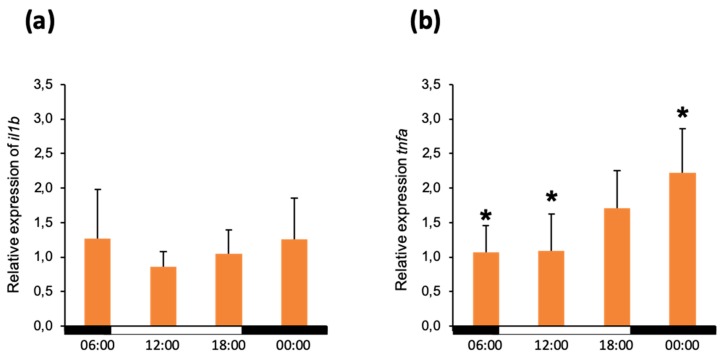
Relative expression of *il1b* (**a**) and *tnfa* (**b**) in the population of head kidney leukocytes following stimulation with inactivated *A. salmonicida*. Cells were incubated for 6 h prior the measurement. The data is presented as mean values with SD error bars. The statistical test used was one-way ANOVA with the Bonferroni *post hoc* test. *p* ≤ 0.1 and statistically significant differences are marked by asterisks (*). The white and black bars on the x-axis demonstrate the dark/light period.

Phagocytosis leads to the activation of downstream pathways resulting in the production of cytokines, orchestrating the onset of the innate immune response. The ability to initiate a fast and effective immune response is vital in determining disease outcome. Thus, we further elucidated whether the time of day has any impact on the capacity of head kidney leukocytes to induce the expression of two key proinflammatory cytokines: IL1β and TNFα. Following the *in vitro* stimulation with inactivated *A. salmonicida*, we evaluated the relative expression of *il1b* and *tnfa*. The results obtained suggested no significant differences between the different sampling times in the relative expression of *il1b* with values ranging between 1.3-fold to 0.7-fold regulation (Figure 3a).

However, we observed a clear trend in the expression of *tnfa*. The lowest values were measured at 6:00 and they increased constantly, having a peak at midnight (Figure 3b). As myeloid cells are the primary source of TNFα, we evaluated the correlation between the percentage of these cells in the head kidney and the relative expression of *tnfa*. The Spearman test of the data provided a ρ (rho) value of 0.8, suggesting a strong correlation between the 2 parameters [28]; however, the *p*-value of the test was not significant, *p* ≥ 0.05.

### 3.4. Recruitment of Leukocytes into the Peritoneal Cavity

**Figure 4:**
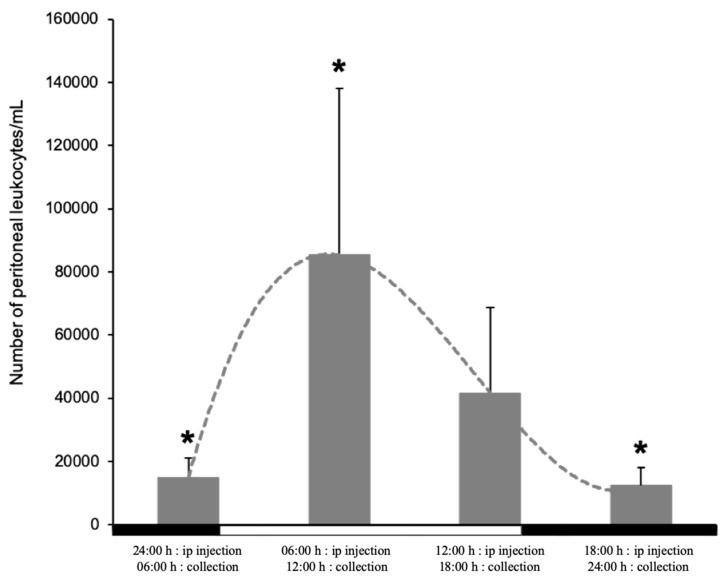
Number of leukocytes recruited to the peritoneal cavity within 6 h post-stimulation with inactivated *A. salmonicida*. Five fish per time point were used for the measurements; the number of cells was estimated from the peritoneal lavage using flow cytometry. Under each bar is detailed the time of the ip injection and the collection time of the peritoneal leukocytes. The data is presented as mean values with SD error bars. The statistical test used was one-way ANOVA with the Bonferroni *post hoc* test. Asterisks (*) represent statistical significance with *p* ≤ 0.05. The white and black bars on the x-axis represent the dark/light period.

Based on the differences in the number of circulating myeloid cells and the time-dependent expression of *tnfa*, we hypothesized that the pace and extent of the inflammatory response will be dependent on the time of day. To further explore this, we used a model of peritoneal inflammation to evaluate the recruitment of leukocytes to the site of inflammation, 6 h post-stimulation with *iA.s*. Obtained results showed significant differences in the number of cells recruited to the inflamed peritoneal cavity during the day. Thus, while the highest number of cells was recruited when fish were stimulated at 6:00 (harvested at 12:00) and 12:00 (harvested at 18:00) (8.5 and 4.1 × 10^4^ cells/mL, respectively), the fish stimulated at 18:00 and 24:00 recruited a considerably lower number of cells (1.4 and 1.2. × 10^4^ cells/mL) (Figure 4).

### 3.5. Bacterial Proliferation in Serum

**Figure 5:**
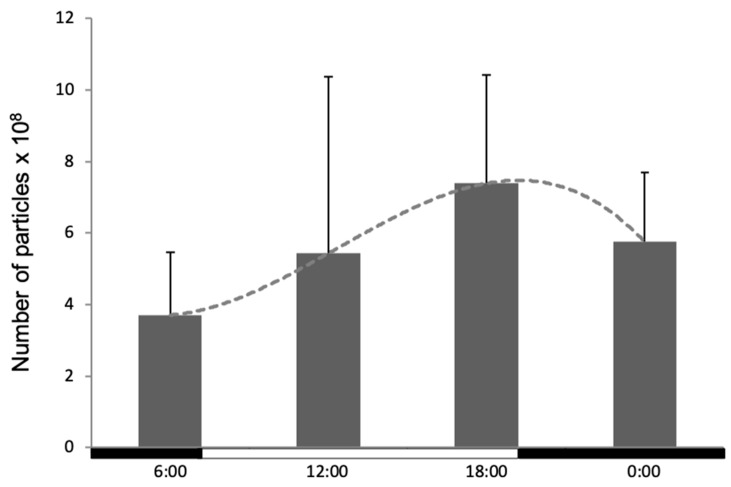
Evaluation of the antimicrobial activity of sera obtained at several time points. Data represent the number of bacteria which grew after a 24-h culture. The data is presented as mean values with SD error bars. The statistical test used was one-way ANOVA with the Bonferroni *post hoc* test considering *p* ≤ 0.05. No statistically significant differences were obtained. The white and black bars on the x-axis represent the dark/light period.

The humoral branch of the innate immune system includes several different proteins, with a known rhythmicity in their production. Since *A. salmonicida* and other pathogens can be detected in the blood of infected fish within hours post-injection, we evaluated the capacity of the sera to limit bacterial growth during the day. For this, *A. salmonicida* was grown for 24 h in the sera isolated at different timepoints. qRT-PCR measurements demonstrated significant differences in the bacteria load in the distinct sera, with the highest number of bacteria observed in the sera obtained in the late afternoon at 18:00 (7.4 × 10^8^ bacteria), while the lowest number was seen at 6:00 (3.7 × 10^8^ bacteria). The number of bacteria observed in the sera from 12:00 and 00:00 reached comparable levels of 5.4 and 5.7 × 10^8^ bacteria, respectively (Figure 5).

## 4. Discussion

Individual immune competence is influenced by external and internal factors. Periodical changes in the light cycle during a day proved to be a large influence on the immune system, controlling more than 8% of the macrophage transcriptome [29]. In mammalian models, circadian rhythms have been previously shown to affect the number of circulating leukocytes, cytokine levels [9,10,11], phagocytic activity [12] and even the course of an infection [18,19]. In fish models, rhythmicity was previously studied with focus on the locomotor activity and feeding behaviour, while its impact on immune competence remains to be explored [5,8]. Here, we aimed to fill this gap and provide a new perspective on the effects of diurnal rhythms on the immune system of rainbow trout.

The composition of peripheral blood leukocytes is routinely used as a marker for the evaluation of immune status during both physiological and pathological conditions. The pioneering works studying the link between daily rhythms and the cell composition in human and rodents described differences between active and rest periods. In humans, the highest lymphocyte counts were observed at night, while counts of monocytes and granulocytes were highest during the day [10,30,31]. In rodents, the opposite trend is observed, with higher number of lymphocytes during the day [32] and higher number of phagocytes in the second half of the dark span [12]. These observations suggest that diurnal rhythms in humans contrasts with nocturnal life in rodents [3,31]. Although the rhythms of salmonids are considered flexible and adjusted to environmental conditions, the 12L-12D light period and regular feeding used in the present study might have induced a level of synchronization [5]. The flow cytometric analysis of the blood and head kidney, identified the myeloid cells as the major population influenced by the time of day. While the IgM^+^ B cells and thrombocytes underwent only mild fluctuations in both investigated niches, the proportion of myeloid cells underwent considerable changes. In blood, the highest number was observed in the early morning and decreased during the day, whereas in head kidney, it followed the opposite trend, being low in the morning and increasing during the day. Considering the function of myeloid cells as the first-line responders, equipped with potent antimicrobial capacities to stop the growth of pathogens and eliminate them, their increased presence in the blood at the beginning of the day points to a higher readiness of the organism to combat potential pathogenic infections. Thus, the rhythms of myeloid cells resemble those of humans rather than rodents [3]. This might correspond not only with the light/dark period, but also with the feeding schedule set on diurnal rhythms. In mammals, the rhythmic recruitment of cells into the blood stream is regulated by the expression of the chemokine receptor CXCR4 and its ligand CXCL12 [33,34]. However, the regulation of this event in teleost fish may involve different players, since the analysis of the expression of these markers revealed no remarkable differences between the investigated time points.

The increased immune competence during the day was further supported by the overall phagocytic capacity of blood leukocytes. The highest ratio of phagocytosis was observed in the early morning hours and declined towards the evening. Assuming diurnal activity, these rhythms resemble those of mice, where the highest phagocytic rate was observed in the dark period [12]. However, in mammals, this process is performed mainly by the professional phagocytic cells, such as dendritic cells, granulocytes, monocytes or macrophages whereas in lower vertebrates, phagocytosis can be accomplished by a variety of cells including B lymphocytes or even thrombocytes [21,35,36]. Indeed, phagocytosis was observed not only in the population of myeloid cells, but also in the subset of B cells and even in thrombocytes. Interestingly, in the blood, B cells were the major population of the phagocytic cells, representing over 50% of phagocytically active cells, while the proportions of myeloid cells and thrombocytes were lower.

Recently, circadian rhythms in rodent models were shown to control transcriptional activity of peritoneal macrophages, the inflammatory response of splenic macrophages and also the recruitment of cells to the inflammation site [29,37]. So far, our data suggest increased immune competence during the day and reduced reactivity during the night, therefore it was intriguing to investigate whether day/night cycles control the expression of proinflammatory cytokines and the recruitment of the cells in the peritonitis model [38]. Surprisingly, using *in vitro* stimulation with inactivated *A. salmonicida*, we identified differences between the investigated cytokines. Thus, while *il1b* showed only minimal rhythmicity, the levels of *tnfa* were significantly dependent on the time of stimulation. This further correlates with the proportion of myeloid cells present in the head kidney prior to stimulation, suggesting that the immune competence of myeloid cells may be independent of their location and time of day. The different capacity of the cells to induce proinflammatory responses following stimulation possibly also contributed to the differences observed in the number of leukocytes attracted to the peritoneal cavity within 6 h post-injection with the inactivated *A. salmonicida*. In agreement with the data observed in a mouse model, where the number of recruited cells was higher during the active period at night [29], the number of peritoneal leukocytes was considerably higher when the fish were stimulated during the light period. However, on the one hand it is important to consider not only the levels of proinflammatory cytokines, but also the presence of the first-responders in the blood, which can serve as a source of neutrophils and other myeloid cells during the acute phase, prior to the mobilization from their reservoirs in the head kidney. On the other hand, we should keep in perspective that pathogens like bacteria and parasites are also regulated by circadian rhythms [39,40,41], and they eventually manipulate the host rhythmicity for their own benefit [42,43] as viruses do as well [44]. Therefore, “when” a pathogen attacks a host, and how the host defend itself, is a fact regulated by an exquisite balance between the species involved.

The overall higher immune competence in the morning was further supported by experiments testing bacterial proliferation in the serum of fish, performed in accordance with previously published experiments [45]. Since bacterial proliferation can be influenced by a plethora of factors including complement activity or concentration of bactericidal peptides such as defensins, hepcidin or cathelicidin, this approach provides valuable information about the ability to prevent potential bacterial spread through the body. The bactericidal activity of the serum showed an increasing trend at the beginning of the active phase and was lower in the late afternoon, however the trend was not statistically significant. Although more experiments must be done to elucidate the nature of this observation, the trend is in agreement with the daily rhythmic serum-mediated bacterial killing activity observed by others [46].

Taken together, the results of the experiment provide compelling evidence connecting daily rhythms to the immune response of an early vertebrate like Rainbow trout. The higher level of immune competence observed during the active phase correlates with the uptake of food and higher activity during the day and it is mediated mainly by the population of myeloid cells. However, it remains to be determined which mechanisms are controlling these differences and how they can be manipulated with the artificial changes in daylight, feeding schedule or possible infections. In addition, it remains an open subject to broaden the research linking day rhythmicity and the adaptive immune responses, driven by T cells and other B cells (non-IgM^+^), that were not addressed in this study due to the limited availability of antibodies. Furthermore, the data presented in this manuscript highlight the relevance of the rhythmicity and its influence on experimental work in the field of fish immunology, inviting interested researchers on the quest to further expand the available knowledge in the field of fish chronoimmunology.
